# The Nexus of Prematurity, Birth Defects, and Intrauterine Growth Restriction: A Role for *Plac1*-Regulated Pathways

**DOI:** 10.3389/fped.2014.00008

**Published:** 2014-02-21

**Authors:** Michael E. Fant, Juan Fuentes, Xiaoyuan Kong, Suzanne Jackman

**Affiliations:** ^1^Department of Pediatrics, Morsani College of Medicine, University of South Florida, Tampa, FL, USA; ^2^Department of Obstetrics and Gynecology, Morsani College of Medicine, University of South Florida, Tampa, FL, USA; ^3^Department of Pathology and Cell Biology, Morsani College of Medicine, University of South Florida, Tampa, FL, USA

**Keywords:** prematurity, Plac1, placenta, birth defects, IUGR, fetal growth

## Abstract

Epidemiological studies have demonstrated an increased prevalence of birth defects and intrauterine growth restriction (IUGR) among infants born prematurely suggesting they share common biological determinants. The identification of key regulatory pathways contributing to this nexus is essential to ongoing efforts to develop effective intervention strategies. *Plac1* is a paternally imprinted and X-linked gene that conforms to this paradigm. Examination of a mutant mouse model has confirmed that *Plac1* is essential for normal placental development and function. Moreover, it is expressed throughout the developing embryo indicating that it also has broad relevance to embryogenesis. Most notably, its absence in the developing embryo is associated with abnormal brain development and an increased risk of lethal, postnatal hydrocephalus identifying it as a novel, X-linked determinant of brain development. The essential and non-redundant roles of *Plac1* in placental and neurological development represent a novel regulatory paradigm for embryonic growth and pregnancy maintenance. Regulatory pathways influenced, in part, by *Plac1* are likely to contribute to the observed nexus of IUGR, prematurity, and birth defects.

## Introduction

In the United States, birth defects and prematurity are the major causes of infant mortality and long-term disability in children (1). Globally, intrauterine growth restriction (IUGR) also emerges as a major determinant of infant mortality and morbidity (2). Together they impose heavy medical, social, and economic burdens on individuals, families, communities, and national development. Often considered as separate entities, increasing evidence suggests they are rooted in common biological pathways. Innovative approaches aimed at reducing their prevalence and associated morbidities will require fundamentally new knowledge regarding the regulatory pathways they share. We will review the evidence supporting their common origins. We will also review a recently identified X-linked gene, *Plac1*, which is essential to both placental and embryonic development and likely contributes to regulatory pathways that link these three outcomes.

## Epidemiological Evidence Linking Prematurity and Structural Birth Defects

Epidemiological studies have demonstrated an unambiguous association between being born prematurely and having a BD (3, 4). In fact, the prevalence of BDs increases progressively as gestational age decreases. The defects most strongly associated with prematurity involve the central nervous system (CNS) (Prevalence Ratio = 16.23), followed by respiratory tract defects (PR = 11.51), cardiovascular defects (PR = 9.29), gastrointestinal defects (PR = 4.98), and genitourinary defects (PR = 4.09). This epidemiological linkage is not surprising. Regulatory interactions required for placental morphogenesis, a fetal tissue *uniquely* required to establish and maintain pregnancy, are also required for the development of other fetal organs. Signaling mechanisms known to be relevant to both embryonic and extra-embryonic development include HuR, Wnt, integrin, RxR, and PPAR-dependent pathways (5–9). Additionally, disruptions in placental function, irrespective of the underlying cause, can secondarily affect embryonic organ development (10, 11). Consequently, disruptions in the placental developmental program are not only likely to increase the probability for pregnancy failure but also likely to influence the development of other fetal organs. While progress has been made in preventing specific BDs and improving pregnancy outcomes complicated by specific maternal conditions, i.e., folate deficiency and diabetes, further progress requires identification of critical pathways that contribute to the “*developmental program*” *shared* by both processes. This intuitively implicates mechanisms underlying the complex spatial and temporal organization that define tissue morphogenesis in both the placenta and embryo.

## Prematurity and Intrauterine Growth Restriction

Compelling evidence has emerged over the last three decades demonstrating that infants delivered prematurely are also disproportionately affected by IUGR. By inference, prematurity and birth defects are part of a continuum that also includes IUGR. Heinonen et al. (12), described a cohort of 120 preterm infants (≤36 weeks) born in the Kuopio province in Finland over a 2-year period, representing 96% of all the preterm births in that region. Birth weight (BW), length (L), and ponderal index (PI) were recorded and IUGR was defined as 2SD below the mean for gestational age for either parameter. IUGR was observed in 41% of the infants when considering at least one growth parameter and a low PI was the most common parameter affected (86%). Additionally, 33% of the infants had more than one growth parameter affected. These observations helped to change the existing pathophysiological paradigm by clinically linking these two entities. The high prevalence of IUGR that was associated with their preterm infants suggested that the sequence of events resulting in their birth actually started weeks earlier, *in utero*, leading to a diminution in growth rate and culminating in early delivery. In other words, the clinical presentation of preterm labor, premature rupture of membranes, or fetal distress represent late or end-terminal stages of a more chronic process.

Subsequent studies in different populations supported these findings. In 2000, Zeitlin et al. (13) reported the results of a case–control study examining the determinants of preterm birth using data from 16 European countries derived from the European Program of Occupational Risks and Pregnancy Outcome (EUROPOP) between 1994 and 1997. A total of 4700 preterm infants born between 22 and 36 weeks of gestation and 6460 control infants born between 37 and 40 weeks were analyzed. Small-for-gestational age (SGA) was defined as a BW below the 10th percentile. Being SGA was found to be significantly associated with preterm birth. Over 40% of preterm births induced for reasons other than the premature rupture of membranes were SGA compared with 10.7% of term infants (OR 6.41). The association was smaller but still significant for preterm births associated with the spontaneous or premature rupture of membranes (OR 1.51). The relationship between growth restriction and prematurity was strongest before 34 weeks gestation. Subsequent clinical studies were consistent with these findings. Doubilet et al. (14) obtained estimates of fetal growth by ultrasound at 24–29.9 weeks gestation and followed the pregnancies until delivery. The measurements of infants delivered very prematurely (24–29.9 weeks) were compared to those of infants delivering at ≥37 weeks. All growth parameters were significantly lower in the group that delivered prematurely compared to those delivering at term. A subsequent study recapitulated these findings. Gardosi (15) obtained ultrasound-derived estimates of fetal weight in a cohort of pregnant women later in gestation (32 weeks). The weights of infants delivering prematurely were compared to those that eventually delivered at term. The weights of term infants displayed a normal distribution whereas the distribution of weights associated with preterm infants were skewed to the left and had a lower median, reflecting a greater proportion of IUGR infants.

The presence of IUGR is underappreciated by most clinicians caring for premature infants unless the infant has been severely affected and is at the extreme end of his/her growth curve. It is important to remain cognizant, however, of the fact that the overall growth (BW, PI, length) trajectory of premature infants is shifted lower, compared to infants delivering at term, even though many have not yet reached the extreme or SGA threshold by the time they deliver. It is clear that a significant portion of preterm infants endure days or weeks of an adverse intrauterine environment that negatively affects their growth and increases their risk for long-term morbidities, independent of being premature.

## Prematurity and the Risk for Cognitive and Psychiatric Impairment

Many studies define birth defects in terms of structurally identifiable anomalies. The March of Dimes, however, has extended this definition to include functional neurological impairments not apparent at birth or associated with identifiable, structural abnormalities (16). This more comprehensive definition of birth defects has been validated by the cumulative weight of several important studies demonstrating an association between prematurity and a variety of functional neurological disorders.

### Psychiatric disorders

Nosarti et al. (17) performed an historical population-based cohort study of all live-born individuals (*N* = 1,301,522) who were registered in the Swedish Medical Birth Register between 1973 and 1985 and living in Sweden at age 16 by 2002. Infants born at 32–36 weeks gestation had a 1.6-fold increase in risk of non-affective psychosis, 1.3× the risk of depressive disorder, and 2.7× the risk of bipolar affective disorder, compared to infants born at term. These risks were higher for infants born earlier than 32 weeks, 2.5×, 2.9×, and 7.9× respectively.

### Attention-deficit hyperactivity disorder

Similarly, Lindström et al. (18) demonstrated an association between preterm birth and an elevated risk for ADHD in Swedish schoolchildren. The children born between 1987 and 2000 (*N* = 1,180,616) who were prescribed ADHD medication between 6 and 19 years of age were identified and linked to their gestational age at birth. There was a significant increase in the use of ADHD medication in children who were born prematurely. Infants born at 23–28 weeks gestation were 2.1× more likely to be taking these medications than children delivered at term. This was followed by 1.6× at 29–32 weeks, 1.4× at 33–34 weeks, 1.3× at 35–36 weeks, and 1.1× at 37–38 weeks. These findings were not affected by genetic, perinatal, or socioeconomic status.

### Autism spectrum disorders

Finally, Johnson et al. (19) conducted a prospective study of all births <26 weeks gestation in the United Kingdom and Ireland in 1995 and found an increased risk for autism spectrum symptoms and autism spectrum disorders (ASD) by middle childhood. Subsequently, by analyzing the medical records of all children ages 0–17 years who resided in Stockholm County from 2001 to 2007 (*N* = 589,114), Abel et al. (20) confirmed the association between ASD and prematurity and further demonstrated an independent effect of fetal growth.

## The Placenta–Brain Axis in Neurological Development

Recently, Bonnin et al. (21) provided compelling evidence for the functional interdependence of the placenta and brain during development that may have relevance to these observations and supports the concept of “premature placental separation” as an independent determinant of brain development. Forebrain circuits are important in ASD, schizophrenia, bipolar disorder, anxiety, and depression. By creating a novel and innovative *ex vivo* mouse model, Bonnin and colleagues were able to demonstrate that the serotonin required for forebrain development was derived exclusively from the placenta between E10.5 and E15.5 after conversion from tryptophan. The hindbrain was not a significant source of forebrain serotonin until after E15.5. Thus, premature separation from the placenta during this time would deprive the developing brain of an essential neurotransmitter during a critical period of development.

## The Placenta as an Experimental Model

**Figure 1:**
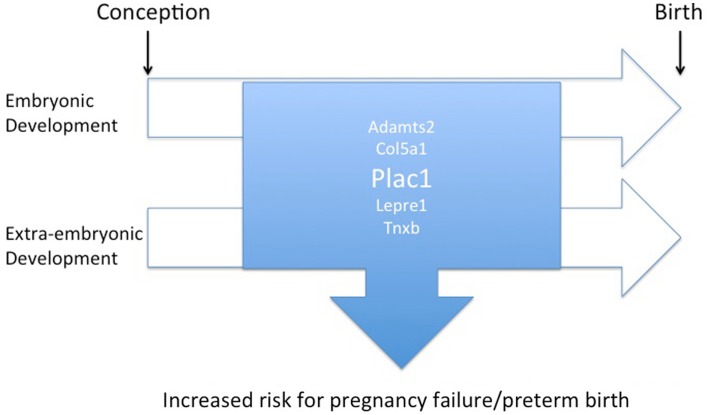
**Diagram illustrating the overlap of regulatory pathways involved with placental and embryonic development that, when disrupted, may contribute to an increased risk of preterm birth or miscarriage as well as birth defects**.

Cumulative evidence derived from epidemiological, clinical, and animal studies provide compelling evidence that prematurity, structural birth defects, and cognitive/psychiatric disorders have roots in shared biological pathways (Figure 1). Identifying and characterizing these pathways and delineating loci that are susceptible to genetic and environmental perturbations are essential for efforts to develop effective intervention strategies aimed at improving outcomes. The placenta, in its unique role of providing an optimal milieu for fetal development, plays an essential role in this regulatory context. *Plac1* (Placenta-specific 1), an X-linked, imprinted gene was recently identified that conforms to this paradigm.

## Identification of the *Plac1* Gene

The *Plac1* gene (placenta-specific 1) was first reported by Cocchia et al. (22). It maps to a region of the X chromosome known to be important for fetal development. Large deletions of the mouse X chromosome, spanning 200–700 kb near the *Plac1* locus, result in fetal growth retardation and neonatal death (23). Further evidence supporting the importance of this locus was provided by Zechner et al. (24) and Hemberger et al. (25). The *Ihpd* (*interspecific hybrid placental dysplasia*) locus maps in proximity to the *Plac1* locus and is associated with abnormal placental development where several X-linked genes may be involved.

Northern analysis and *in situ* hybridization indicated *Plac1* expression is restricted primarily to cells of trophoblastic lineage (22, 26) hence its name *Plac*enta-specific *1*. However, recent studies (using qRT-PCR) have demonstrated expression in multiple embryonic tissues (1–10% of placental expression). Human *PLAC1* expression is tightly linked to trophoblast differentiation and localizes to the apical region of the cytosolic compartment of the syncytium in proximity to the maternal-facing, microvillus brush border membrane, consistent with function at or near the maternal–fetal interface (27, 28).

## *Plac1* in Placental Development

**Figure 2:**
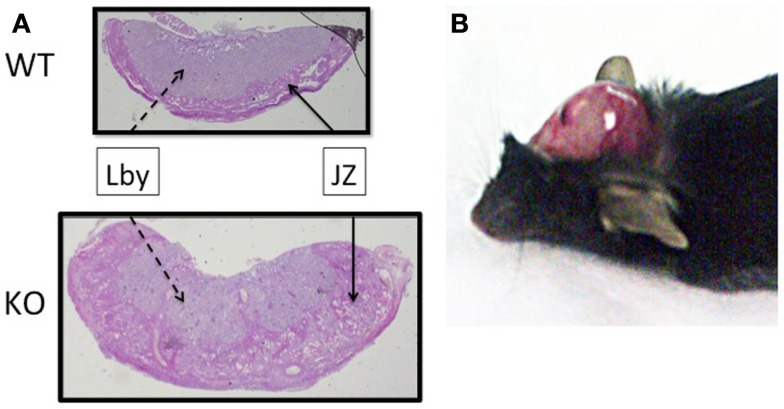
**Effect of *Plac1* ablation on placental and brain development**. **(A)** H&E stain of E16.5 mouse placentae associated with KO and WT males. JZ, junctional zone; Lby, labyrinth. **(B)** Postmortem, 6-week-old X^m−^X Het female with hydrocephalus. The cranium was retracted to expose the brain tissue.

Using a mutant mouse model, we have confirmed that *Plac1* is essential for normal placental development (29). *Plac1* ablation results in placentomegaly and IUGR. At E16.5, knockout (KO) and heterozygous (Het) placentae deriving the *Plac1*-null allele from the mother (X^m−^X) weigh approximately 100% more than WT placentae whereas the corresponding embryos weigh 7–12% less. Histologically, the *Plac1* mutants exhibit an expanded junctional zone that migrates into the labyrinth where maternal–fetal transport takes place (Figure 2A). By contrast, Het placentae that derive the null allele from the father (XX^p−^) exhibit normal growth and are histologically indistinguishable from WT placentae consistent with paternal imprinting. While *Plac1* ablation does not result in an obligatory lethal phenotype it does reduce postnatal viability of nullizygous offspring.

Observations from human studies have provided evidence that *PLAC1* is relevant to the establishment and maintenance of pregnancy by identifying it as a potential biomarker for gestational pathologies. We reported that women can become sensitized to the PLAC1 antigen during pregnancy and the presence of anti-PLAC1 antibodies may be associated with infertility and/or recurrent pregnancy loss (30). This observation was confirmed by Matteo et al. (31) who demonstrated increased anti-PLAC1 antibody titers in women with a history of infertility. Additionally, Farina et al. (32) and Concu et al. (33) demonstrated that *PLAC1* mRNA can be detected in maternal serum as early as 8 weeks gestation and throughout pregnancy. Circulating *PLAC1* mRNA is markedly diminished in pregnancies associated with vaginal bleeding prior to 20 weeks gestation. Subsequent studies demonstrated elevated levels of circulating *PLAC1* mRNA in pre-eclampsia that were directly related to disease severity (34, 35). These findings are consistent with a role for *PLAC1* in pregnancy maintenance and possibly as a useful biomarker of gestational pathologies.

Although there is no experimental evidence directly linking *PLAC1* to preterm delivery, it does appear to disrupt placental development in a manner likely to interfere with its ability to adapt to the physiological and environmental challenges of pregnancy, thereby predisposing the pregnancy to early failure. There is some evidence linking genes expressed at the maternal–fetal interface with the maintenance of pregnancy. In a recent review, Anum et al. (36) identified mutations in several genes (COL5A1, COL5A2, COL3A1, COL1A1, COL1A2, TNXB, PLOD1, ADAMTS2, CRTAP, LEPRE1, and ZMPSTE24) associated with connective tissue disorders involving multiple organs, i.e., Ehlers–Danlos syndrome, osteogenesis imperfecta, and restrictive dermopathy that were also associated with prematurity, presumably resulting from the premature rupture of membranes.

## *Plac1* in Brain Development

While characterizing the mutant mouse model we unexpectedly discovered that *Plac1* is also expressed throughout the developing embryo where it plays a major role in brain development (37). *Plac1* ablation is associated with a 22 and 11% increased risk for lethal hydrocephalus (HC) and other brain abnormalities in KO males (X^m−^Y) and Het females inheriting the null allele from the mother (X^m−^X), respectively (Figure 2B). By contrast, Het females inheriting the null allele from the father (XX^p−^) do not develop HC, consistent with paternal imprinting. These observations established *Plac1* as a novel, X-linked determinant of brain development.

The X chromosome is enriched for genes involved in brain development and cognitive function and several are clustered around Xq26.3 (38, 39), where human *PLAC1* localizes. Families carrying microdeletions or duplications in this region (encompassing *PLAC1*) report family members with a variety of CNS defects including reduced cognitive function, microcephaly, and neural tube defects (40–43). Our experimental findings indicate *PLAC1* is one of the genes at this locus (Xq26.3) that is essential for normal brain development and likely contributes to the clinical abnormalities observed in the reported families.

The development of HC was the first clue that *Plac1* was involved in mouse brain development. At least 43 genetic loci linked to hereditary HC have been identified in animal models and humans, irrespective of the presence of other associated defects (44, 45). In fact, *X-linked* HC is the most common form of hereditary HC accounting for 15–25% of primary idiopathic HC in newborn males (46–49). A variety of CNS malformations and clinical symptoms have been reported in humans with X-linked HC including ataxia, reduced cognitive function, and gait abnormalities. Linkage and pedigree analysis led to the discovery of the neural cell adhesion molecule, *L1CAM*, as the genetic basis for most of the cases of this disorder (50). Mouse models of *L1cam* mutations recapitulated the phenotypic variability observed in humans, including the absence of overt HC in some cases, pointing to interactions with other genetic or environmental factors (51–53). Cases of X-linked HC, not associated with mutations in *L1CAM*, have also been reported. A family with adult onset X-linked HC has also been described (54), characterized by normal pressure hydrocephalus (NPH), seizures, mental disorganization, behavior problems, and urinary incontinence. Although no gene locus has been identified, its transmission is consistent with X chromosome linkage. Many of the genetic determinants of developmental brain disorders have yet to be identified. These studies underscore the importance of the X chromosome, particularly the *PLAC1* locus, to brain development and neurological function.

## *Plac1* Protein Function

While nothing is known regarding the functional properties of *Plac1*, inferences relevant to tissue morphogenesis can be made based on its sequence. The human ORF encodes a putative protein of 212 amino acids whereas the mouse encodes a 173 amino acid product. Both contain a cleavable signal peptide and are predicted to exist as extracellular peptides. Significant sequence homology (approximately 30%) exists between *Plac1* and the zona pellucida 3 (ZP3) protein, a sperm-binding glycoprotein important for fertilization. The zona pellucida domain (ZPD) defines a conserved family of membrane-anchored matrix proteins having important roles in the localized organization of epidermal cell membranes to influence organ development and plasticity (55–61). Therefore, a key feature of *Plac1*’s function is likely the modulation and integration of signaling pathways linking the cell membrane to the extracellular environment to alter cell shape, motility, and plasticity during morphogenesis.

## Summary

Recognition of the paradigm linking IUGR, prematurity, and birth defects to common biological substrates will facilitate efforts to understand their root causes. The identification of key regulatory pathways contributing to this nexus will provide important knowledge necessary to enhance efforts to prevent, diagnose, and intervene in troubled pregnancies and affected offspring. *Plac1*, an X-linked, imprinted gene with unique relevance to placental and embryonic development, is a novel candidate gene conforming to this paradigm. Defining its developmental role will provide important new insights into embryonic development and pregnancy maintenance.

## Author Contributions

Michael E. Fant conceived the work and contributed to its design, data analysis, and interpretation and wrote the initial draft of the manuscript. Juan Fuentes contributed to the acquisition, analysis, and interpretation of data and was involved in critically reviewing the manuscript for intellectual content. Xiaoyuan Kong contributed to the acquisition, analysis, and interpretation of data as well as critically reviewing the manuscript. Suzanne Jackman contributed to the acquisition, analysis, and interpretation of data and critically reviewed and revised the manuscript. All authors approved the final version submitted for publication.

## Conflict of Interest Statement

The authors declare that the research was conducted in the absence of any commercial or financial relationships that could be construed as a potential conflict of interest.
